# Hypertrophic Osteoarthropathy: A Secondary Manifestation of Malignant Melanoma

**DOI:** 10.1155/2021/6691320

**Published:** 2021-01-18

**Authors:** Shiva Malaty, Aditya Gupta

**Affiliations:** HonorHealth Internal Medicine Residency, Scottsdale, AZ, USA

## Abstract

**Background:**

Hypertrophic osteoarthropathy (HOA) is a rare finding in the setting of metastatic melanoma. A majority of cases of secondary HOA involve lung malignancies. Evaluation of presenting symptoms such as polyarthralgia and clubbing followed by review of imaging studies are diagnostic steps for HOA. *Case Presentation*. We present a 60-year-old female with a history of metastatic melanoma who presented with bilateral and symmetric polyarthralgia and clubbing. A plain film radiograph demonstrated periosteal thickening involving the metacarpals and proximal phalanges as well as the distal radius and ulna, consistent with HOA. The patient was treated with nonsteroidal anti-inflammatory agents for supported care.

**Conclusion:**

HOA may be a secondary manifestation of metastatic melanoma. Recognition and supportive care of this condition may lead to improved quality of life for patients.

## 1. Introduction

Hypertrophic osteoarthropathy (HOA) is characterized by digital clubbing, periostosis, and arthralgia of extremities. This condition is secondary to abnormal proliferation of osseous and soft tissue. There are two forms of HOA: a primary genetic form, which accounts for approximately 3% of cases, and a secondary form, mainly attributed to paraneoplastic syndrome from non-small cell lung cancer and to a lesser degree, from pulmonary diseases such as sarcoidosis, pulmonary tuberculosis, and cystic fibrosis [1, 2]. The proposed pathophysiology involves the release of platelet-derived growth factor and vascular endothelium-derived growth factor [3]. These growth factors lead to increased bone production and vascular hyperplasia, which leads to clinical signs of digital clubbing.

## 2. Case Presentation

**Figure 1:**
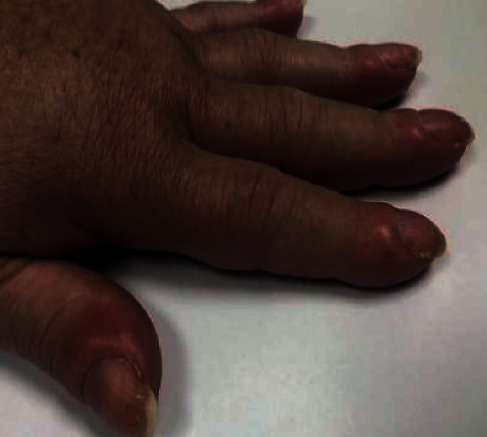
Clubbing of distal phalanges.

**Figure 2:**
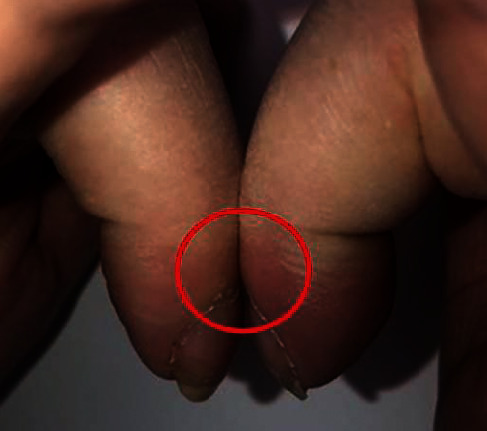
Schamroth sign, disappearance of normally appearing diamond-shaped window between dorsal pads of opposite terminal phalanges.

We present the case of a 60-year-old female with a medical history of B-RAF wild-type metastatic melanoma with metastasis to the liver and lung, presenting with swelling over the fingers and toes. The patient reported the presence of edema for years and was told it was related to an autoimmune condition given a previously positive ANA result. The patient stated that the swelling was also associated with symmetric polyarthralgia of the upper and lower extremities. She denied any alleviating or aggravating factors. On physical exam, clubbing was present on bilateral distal phalanges (Figure 1). Additionally, when placing the pads of bilateral terminal phalanges in dorsal opposition, disappearance of the normal diamond-shaped window was found, indicating a positive Schamroth sign (Figure 2). The patient underwent lab work, which revealed an alkaline phosphatase level of 146.

**Figure 3:**
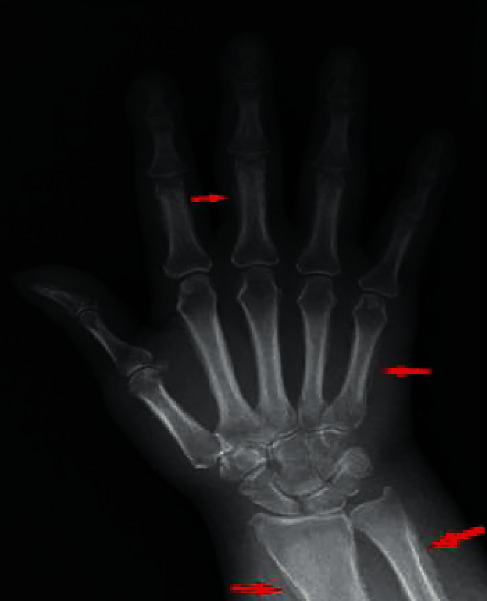
Periosteal thickening involving the metacarpals, proximal phalanges, distal radius, and ulna.

Plain film radiography of the upper extremities demonstrated periosteal thickening involving the metacarpals and proximal phalanges as well as the distal radius and ulna, consistent with hypertrophic osteoarthropathy (Figure 3). Given patient's terminal malignancy, no further oncological or surgical treatments were available. The patient was started on a nonsteroidal anti-inflammatory agent, ibuprofen, for symptomatic relief. She demonstrated significant improvement of arthralgias during her three-week follow-up. The patient went on to hospice care and shortly passed.

## 3. Discussion

The proposed pathophysiology of HOA involves the release of platelet-derived and vascular endothelium-derived growth factors. The exact mechanism of disease in HOA is uncertain. This diagnosis should be considered in the setting of clubbing with imaging findings supportive of periosteal thickening. In the setting of HOA with no underlying cause, a plain film radiograph of the chest should be indicated to rule out pulmonary etiology [3].

There are no specific or reliable lab tests available for the diagnosis of HOA. A plain radiograph is the most telling diagnostic modality for HOA. The presence of periosteal thickening is the most specific radiographic finding associated with HOA. This finding may be generalized to include more than one specific region of the bone or localized to a specific site of the bone [4, 5].

Treatment of the underlying disease process is key for secondary HOA. Supportive care with nonsteroidal anti-inflammatory agents as well as bisphosphonates may provide relief. VEGF inhibitors have also been proposed as therapeutic options for alleviating pain symptoms in secondary HOA [6].

This case exhibits the importance of both, a thorough clinical history and physical examination. This diagnosis should be considered in patients presenting with polyarthralgia and appropriate imaging findings. Early diagnosis of this condition combined with appropriate management and therapy may result in improved quality of life for affected patients.

## Data Availability

All data in this case report are taken from the clinical records.
